# Molecular Research on Huntington’s Disease

**DOI:** 10.3390/ijms24054310

**Published:** 2023-02-21

**Authors:** Luis M. Valor

**Affiliations:** Unidad de Investigación, Hospital General Universitario Dr. Balmis, ISABIAL, 03010 Alicante, Spain; valor_lui@isabial.es; Tel.: +34-965-913-988

Huntington’s disease (HD) is a devastating neurodegenerative disorder caused by an aberrant expansion of CAG triplets in the *HTT* (Huntingtin) gene. This mutation leads to the production of a toxic peptide (mHTT) and the loss of a functional allele that is behind pleiotropic symptomatology that progressively compromises the motor, cognitive, affective and mental capabilities of patients until final decease. Apart from the nervous system impairment, the HTT protein is ubiquitously expressed and, despite not being fully acknowledged until recent times, peripheral organs (e.g., liver, pancreas, heart, spleen) and cells (e.g., myeloid and lymphoid lineages) also exhibit certain malfunction, contributing to the plethora of pathological signs and potentially providing general principles regarding the molecular pathogenesis in the disease, as reviewed by Ana I. Arroba and colleagues [1]. 

Despite knowing the cause of the disease more than thirty years ago, neither a cure nor an effective treatment is available, and this is due to the complex alterations (and interactions) between multiple cellular processes. This is well exemplified in [2] in which Hoon Ryu and collaborators provide a succinct overview of the molecular alterations provoked by the CAG expansion in the *HTT* gene at the level of epigenetics, vesicle trafficking and axonal transport, mitochondria and mitophagy. This review also reminds us that not only neurons but also astrocytes and oligodendocytes can be affected by the ubiquitous expression of mHTT, interfering with their roles in neuronal homeostasis and synapse transmission and exacerbating neuronal dysfunction. Meanwhile improved formulations of antisense oligonucleotides targeting HTT are still under development, it is worth exploring alternative therapeutic approaches aimed at restoring defective gene expression, mitochondrial function and autophagy or at preventing mHTT aggregation and accumulation and neuronal death, among others [2]. Interestingly, the use of the invertebrate model can accelerate the identification of potential therapeutic targets in systematic screens, for example, by examining the effects of RNA interference assays in a *C. elegans* model for HD, as documented by Jeremy M. Van Raamsdonk’s team. In this example, targeting genes involved in mitochondrial fission can rescue mitochondrial fragmentation in neurons and can restore movement and life span in HD worms [3]. As a complementary approach consisting of the application of novel bioinformatics tools, deciphering and understanding the network of protein–protein interactions surrounding the scaffolding protein HTT can open new lines of research by providing modulators of HD as potential drug targets for future studies [4]. 

Therapeutic administration requires the use of biomarkers to discern the real stage and rate of progression of the disease in each patient. We reviewed that HTT and its mutant form, markers of neuronal injury (e.g., NF-L), the phosphodiesterase PDE10A, the neuropeptide Y, oxidative stress markers, transcriptional deregulated genes in blood cells, inflammatory markers, genetic modifiers (polymorphisms), etc., are prominent biomarkers which suggest that their potential applicability into clinics is promising but still challenging [5]. Genetic modifiers deserve further attention due to their special relevance in the modulation of the HD ethiopathogeny, and Jakob von Engelhardt and coworkers illustrate this fact in HD mouse strains, as they can exhibit different pathophenotypical traits, including neuronal electrophysiology properties, as a result of the still obscure interplay between *HTT* mutation and the genetic background [6]. 

In this Special Issue, we provide some glimpses of the molecular research that is being conducted in HD but several fronts of research are simultaneously open. Despite the vast cumulated knowledge and recent advances, we still need in-depth characterizations of the mechanisms involved in this pathology, as patients are still orphans for biomarkers and therapies in their clinical management. We hope that the advent of new technologies that are increasingly accessible to worldwide researchers, fueled by current policies for data sharing and open access, will provide effective solutions in the next future.
